# Generation and propagation of a sine-azimuthal wavefront modulated Gaussian beam

**DOI:** 10.1038/srep30032

**Published:** 2016-07-21

**Authors:** Guanming Lao, Zhaohui Zhang, Meilan Luo, Daomu Zhao

**Affiliations:** 1 Department of Physics, Zhejiang University, Hangzhou 310027, China

## Abstract

We introduce a method for modulating the Gaussian beam by means of sine-azimuthal wavefront and carry out the experimental generation. The analytical propagation formula of such a beam passing through a paraxial ABCD optical system is derived, by which the intensity properties of the sine-azimuthal wavefront modulated Gaussian (SWMG) beam are examined both theoretically and experimentally. Both of the experimental and theoretical results show that the SWMG beam goes through the process from beam splitting to a Gaussian-like profile, which is closely determined by the phase factor and the propagation distance. Appropriate phase factor and short distance are helpful for the splitting of beam. However, in the cases of large phase factor and focal plane, the intensity distributions tend to take a Gaussian form. Such unique features may be of importance in particle trapping and medical applications.

Up to now, a considerable attention has been paid to the interaction of laser beams and materials. However, many technologies involving laser welding, laser processing, and laser medical applications are susceptible to localized overheating caused by the uneven energy distribution of laser beams. Hence, it is of necessity and importance to shape the laser beams. In some circumstances it is desirable to have a laser beam with a different irradiance distribution from the one originally coming out of the laser cavity. In particular, it is often required to have a uniform intensity distribution over the cross section of the beam to avoid material damage^1^. During the past decades, laser beam shaping has attracted more and more attention due to their wide applications in various fields, such as materials processing, medical applications, optical data storage and laser printing^2^,^3^. The ability to shape the phase and amplitude of a laser beam has been recognized since the early days of laser physics and the full scope of optical components has been applied to beam shaping.

It is also worth pointing out that many approaches have been proposed for the purpose of generating some new kinds of laser beams whose profiles differ from those of classic Gaussian modes. Among them are the Airy beams, Bessel-Gaussian beams, super-Gaussian beams, sinusoidal Gaussian beams, flat-topped beams, Laguerre-Gaussian beams, and anomalous vortex beams^4^,^5^,^6^,^7^,^8^,^9^,^10^. A demand for these beams is motivated by the increase in the number of their potential applications and, in particular, by the development of new high power laser sources and systems. The most typical method is using the diffractive techniques, which are based on a spatial light modulator (SLM). These techniques are more interesting and popular because they have the advantage of providing dynamic and programmable modulations. Moreover, so far no SLM exists in which the phase and the amplitude of a light wave can be simultaneously modulated. That is, most of the developed techniques codify a complex field by the amplitude modulation or phase modulation exclusively. Therefore, in this paper we report the experimental generation of a sine-azimuthal wavefront modulated Gaussian (SWMG) beam on the basis of phase modulation and investigate its propagation properties through free space or a focusing system.

## Results

It is assumed that the electric field of the SWMG beams in the source plane has a form of:


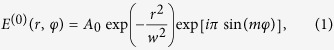


where *A*_0_ and *w* refer, respectively, to the characteristic amplitude and waist width of the incident beam, *φ* denotes the azimuth angle with *m* being the phase factor. For the sake of convenience, *A*_0_ equals to 1.

Within the framework of the paraxial approximation, the electric field in the transverse plane z = const > 0 through an *ABCD* optical system can be studied with the help of the generalized Collins formula^11^,^12^.


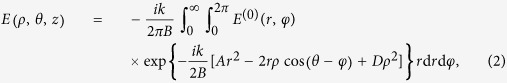


where *A*, *B*, *C* and *D* denote the elements of the transfer matrix of the optical system, *k* is the wave number. And in Eq. (2), the phase term exp(*ikL*_0_) along the axis between the two reference planes has been omitted.

The final analytical expression of the electric field in the output plane is


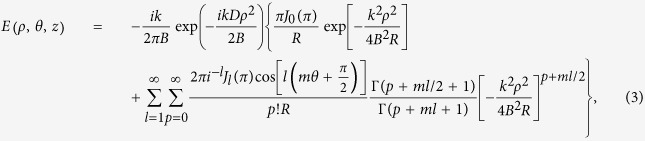


where 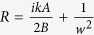.

### Propagation of a SWMG beam in free space

The transfer matrix of distance *z* reads as^12^.


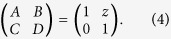


On substituting Eq. (4) into Eq. (3), it reduces to


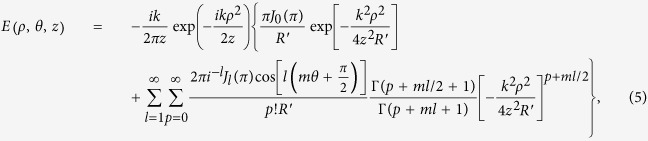


where 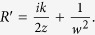


**Figure 1:**
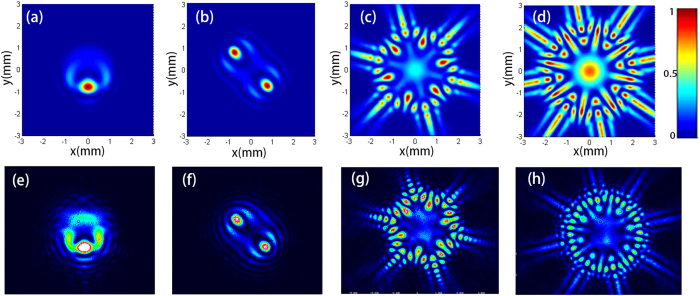
Theoretical (**a**–**d**) and experimental (**e**–**h**) results of the intensity distributions of the SWMG beam with different values of phase factors when propagating in free space for *z* = lm. (**a**,**e**) *m* = 1; (**b**,**f**) *m* = 2; (**c**,**g**) *m* = 6; (**d**,**h**) *m* = 8.

Distributions of the intensity illuminated by a SWMG beam at propagation distance *z* = 1 m in the free space are theoretically and experimentally shown in Fig. 1, where the dependencies on the phase factors *m* are illustrated. Fig. 1(e–h) represent the experimental results, which are well consistent with the theoretical calculation results shown in Fig. 1(a–d). From these figures, one finds that the intensity of the SWMG beam is closely determined by *m*. In the case of *m* = 1, the pattern of the intensity is represented by an asymmetric ring and the area of bright intensity moves slightly away from the optical axis. In Fig. 1(b,f), it evolves into two intensity spots, which are located in the diagonal. As we increase the phase factor continually, a lot of intensity spots extend outside around this center. And for large phase factor (*m* = 8), the center spot is gradually formed. Therefore, modulating the parameter *m* may provide us a convenient way for shaping the beam profile, which is useful in the areas of particle trapping and optical manipulation. The movement of the intensity may be helpful for propelling the nanoparticles. The dark zone of the beam is required for trapping particles with refractive index smaller than that of the ambient, and a Gaussian beam can be employed to trap particles with refractive index larger than that of the ambient^13^,^14^,^15^.

**Figure 2:**
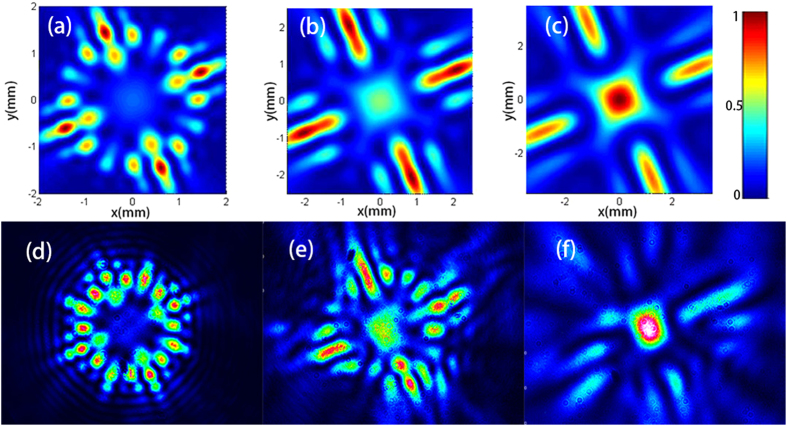
Theoretical (**a**–**c**) and experimental (**d**–**f**) results of the intensity distribution of the SWMG beam with *m* = 4 on propagation in free space at different distances. (**a**,**d**) *z* = lm; (**b**,**e**) *z* = 2 m; (**c,f**) *z* = 4 m.

The intensity evolution of a SWMG beam at several certain propagation distances is explored by taking the case of *m* = 4 as an example, as shown in Fig. 2. Similar interesting features are exhibited on propagation, which is distinctively different from that of the source plane, where the profile of the intensity takes a Gaussian form. When the beam propagates a relatively short distance, there is a hollow intensity profile with four maximum intensity lines outside, which are located in the radial direction. In addition, two weak intensity spots appear beside each line [see Fig. 2(a,d)]. With the increase of propagation distance, there occurs one intensity spot at the center, and the secondary maximum outside becomes weaker and weaker. Finally, the energy is focused on the center [see Fig. 2(c,f)]. It should be noticed that the experimental measure of the intensity shown in the circumstance of *z* = 2 m and *z* = 4 m are reflected by the mirror once and twice, respectively, hence there is energy loss at the edge [see Fig. 2(e,f)].

### The focusing properties of a SWMG beam

As a particular example, we will now consider the focusing properties of the SWMG beam, the corresponding transfer matrix between the source plane and the observed plane in the focusing system is^12^


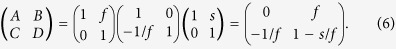


On substituting Eq. (6) into Eq. (3), we obtain the following expression for the electric field of the SWMG beam in the focused plane:


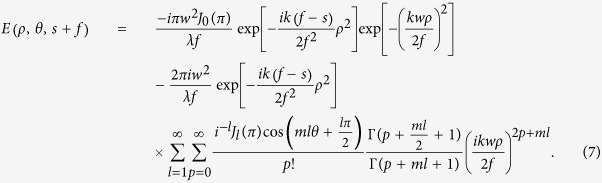


**Figure 3:**
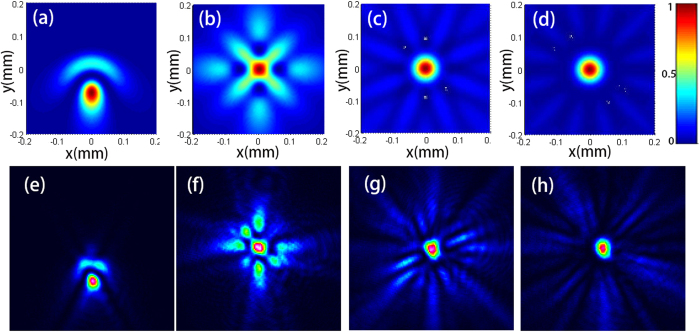
Theoretical (**a**–**d**) and experimental (**e**–**h**) results of the intensity distribution of a SWMG beam with different values of phase factor in the geometrical focal plane. (**a**,**e**) *m* = 1; (**b**,**f**) *m* = 2; (**c**,**g**) *m* = 4; (**d**,**h**) *m* = 6.

The focused intensity distributions of the SWMG beam versus the phase factors are displayed in Fig. 3. One can find from Fig. 3(a) that the focal pattern is formed by one spot accompanied by a low intensity arc. When we increase *m* up to 2, there occurs one cross shape focal pattern, and four weak intensity spots appear between the adjacent branches of the center cross shape. As the phase factor increases, the whole focal pattern is one intensity spot in the center and it just looks like a Gaussian form, which is consistent with Fig. 2.

## Discussion

### Some extreme cases

We can also analyze the central intensity distribution of a SWMG beam with extremely large phase factor by investigating the property of the Eq. (5). When *m* ≫ 1, if we only consider the central part of the pattern, we can find that the second term of the Eq. (5) could be eliminated. Since the minimum value of *ml* is *m*, according to the Stirling’s formula 
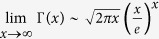
, we can rewrite part of this term as follows:


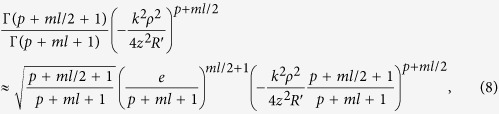


where *p* ≥ 0, *l* ≥ 1 , and 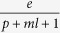 is much smaller than 1. Thus, if *ρ* is small enough, 
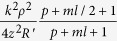
 could be also small enough so that the second term in Eq. (5) could be eliminated. As a result, the electric field of a SWMG beam with large phase factor under the paraxial condition could be described as


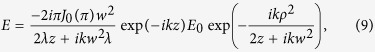


hence the central intensity distribution becomes.


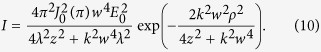


Moreover, for the situation of *m* ≥ 4, when the propagation distance is great enough (*z* ≫ 1 m), define 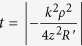 and then we can know that *t* ≪ 1. Since the minimum value of *ml* is *m*, if *m* ≥ 4, the second term of the Eq. (5) would be a high order infinitesimal O(*t*) comparing to the first term. As a result, the electric field of this situation has the same form as Eq. (9) if we omit the second term, and its central intensity distribution could be also approximately described by Eq. (10).

### Error analysis

In order to illustrate more properly that the experimental results agree well with the theory, we choose the case of *m* = 2 in free space, which is illustrated in Fig. 1(b,f), for interpretation. Since the two maximal intensity points distribute on a diagonal line in Fig 1(b,f), we select the diagonal line as an axis, and the mid point of the two points as its origin. In addition, we assume the distance between the two maximal intensity points as 1 in order to eliminate the size discrepancy of Fig. 1(b,f), that is, the relative distance between the origin and a maximal intensity point is 0.5. If we set the direction of the axis as 
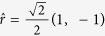
 on the output plane, the location of the points on the axis could be described by relative location, and the relative location of the maximal intensity points are ±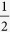.

**Figure 4:**
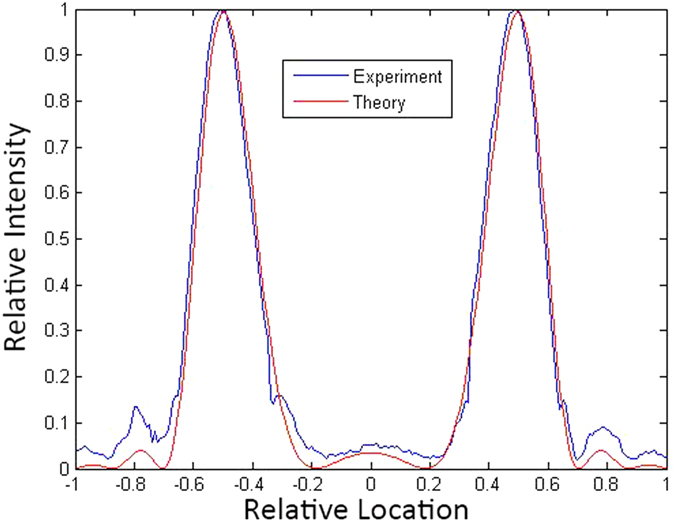
The profile of the experimental and theoretical relative intensity of the SWMG beam with phase factor *m* = 2 and propagation distance *z* = 1 m.

The relationships between the relative intensity of SWMG beam and the relative location of the points on the axis are shown in Fig. 4. In this figure, we can see that the profile of the experimental relative intensity distribution agrees well with the theory when the relative intensity is greater than 0.3. Because of the errors produced by the CCD, the experimental curve is no longer smooth and discrepancy shows up when the relative intensity is lower than 0.2. However, experimental relative locations of the maximum points are well forecasted by the theoretical curve, with a maximum deviation about 0.05. In this aspect, we can see that Eq. (5) could not only calculate the intensity distribution pattern of the SWMG beam in free space, but also forecast the profile of the distribution and the location of the maximum intensity points well.

However, the profile of the experimental intensity distribution of the focused system is a little different from the theoretical study. That is due to three following reasons. The first reason is that the lens is not absolutely vertical to the optical axis, which could lead to the deformation of the intensity distribution; the second one is that the CCD is slightly deviated from the focal point; and the third one is that the pattern is too small (the width of the intensity spot is about 0.1 mm) for the CCD to measure the intensity distribution with enough accuracy.

## Methods

### Theoretical analysis

On substituting from Eq. (1) into Eq. (2), it follows that:


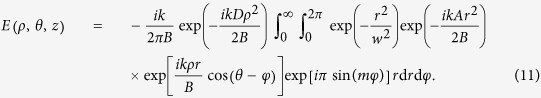


In order to determine this phase modulation we employ the Jacoby-Anger identity to express the hologram transmittance by its Fourier series.


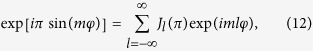


where *J*_*l*_(.) represents the *l*th integer order Bessel function of the first kind.

And using the following formulas^16^


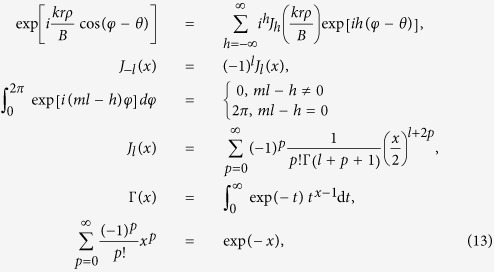


where Γ(*x*) is the Gamma function.

On substituting from Eqs. (12) and (13) into Eq. (11) and taking the complex integrations, one finds the final analytical expression of the electric field in the output plane could be described by Eq. (3).

### Experimental generation of a SWMG beam

There are two ways which could be used for generating the SWMG beam. The first one is using a phase plate^17^ which could impose a sine-azimuthal retardation on the optical field. It is a transparent plate with refractive index *n* and its height is proportional to sin(*mφ*), where *φ* is the azimuthal angle and *m* is the phase factor. That is, the height of the plate could be expressed by the following formula:


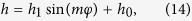


where *h*_1_ is the step height and *h*_0_ is the base height of the device. As a result, the azimuthal optical phase delay should yields the following equation:


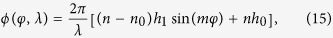


where *n*_0_ is the refractive index of the surrounding medium.

**Figure 5:**
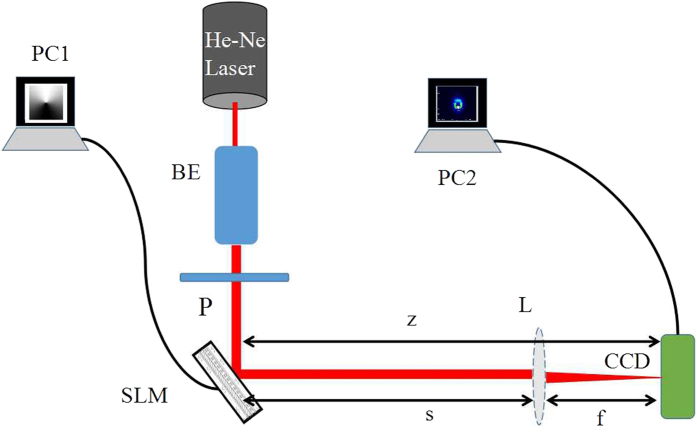
Experimental setup for generating a SWMG beam and measuring its intensity in free space and focusing system. BE, beam expander; P, polarizer; SLM, spatial light modulator; L, thin lens; PC1, PC2, personal computer.

**Figure 6:**
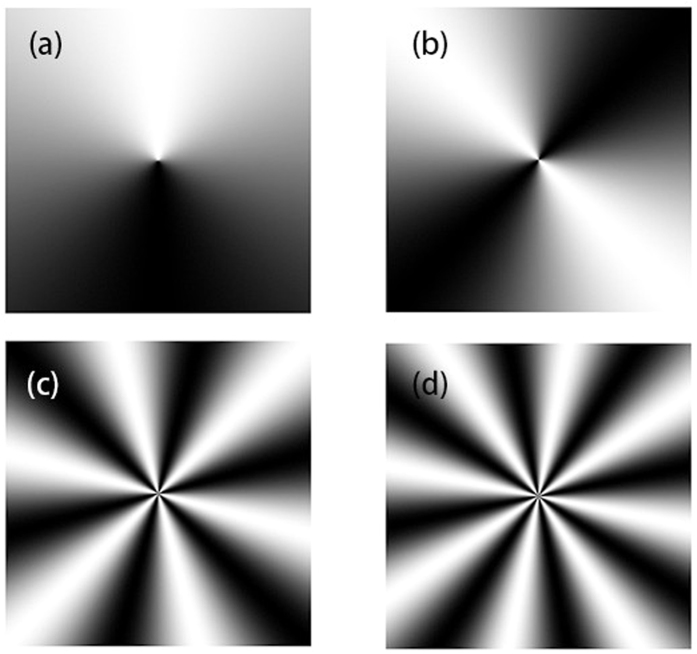
The holographs for generating the SWMG beam with different phase factors. (**a**) *m* = 1; (**b**) *m* = 2; (**c**) *m* = 6; (**d**) *m* = 8.

The second way is using the spatial light modulator (SLM), which is used in our experiments. Transformation of a laser beam into an arbitrary complex field can be easily performed by means of phase modulation of SLM. This way is of versatility and robustness, which facilitates its accurate implementation. In this section we will report about the generation of a typical SWMG beam and carry out experimental study of its intensity properties to test the above theoretical results. The experimental setup for generating the SWMG beams and measuring its intensity through free space and a focused system is shown in Fig. 5. The straightforward way is to modulate the sine-azimuthal phase into the Gaussian beam, in such a way as to create a change in the intensity of the laser beam. In our scheme, the reflective phase SLM is illuminated by a linearly polarized He–Ne laser beam with *λ* = 633 nm that is conditioned by a beam expander. The transmitted beam just behind the SLM is set as the generated SWMG beam source, of which the transverse beam width is controlled to be 1 mm. A charge-coupled device (CCD) is placed on the optical axis and the distance between the SLM and itself is *z* when the free space propagation properties are investigated. In order to study its focusing properties, a thin lens with focal length *f* = 300 mm is seated before the CCD, which is located at the focal plane. A personal computer (PC2) connects the CCD and is used to measure the focused intensity. The separation between the SLM and the thin lens is *s*. To avoid the first surface reflection in the SLM, we select the first-order diffraction pattern of the beam from the SLM. The patterns of the holograph for generating a SWMG beam of different phase factors are displayed in Fig. 6.

## Conclusions

In conclusion, a new beam source named the SWMG beam has been introduced and its analytical propagation formula passing through a paraxial ABCD optical system is derived. On that basis, we investigated the intensity distribution of such a beam on propagation in free space and the focused system, respectively. Furthermore, we also have experimentally generated a SWMG beam and carried out the experimental measures of the intensity, which verifies that our experimental results agree well with the theoretical predictions. Results show that the SWMG beam displays unique features on intensity properties, varying from beam splitting to Gaussian-like profile. And such of changes can be realized by adjusting the phase factor. In addition, the evolution process is also determined by the propagation distance. At the relatively short distances, this beam will split. While in the far field (or in the focal plane), the energy of the beam will concentrate to the center and the intensity distribution will evolve into having Gaussian form. Due to this kind of unique propagation properties, the SWMG beam may find applications in optical manipulation, medical applications and material thermal processing.

## Additional Information

**How to cite this article**: Lao, G. *et al*. Generation and propagation of a sine-azimuthal wavefront modulated Gaussian beam. *Sci. Rep.* **6**, 30032; doi: 10.1038/srep30032 (2016).
